# Electrophysiological Mechanisms of Ventricular Fibrillation Induction

**Published:** 2005-01-01

**Authors:** Nipon Chattipakorn, Kirkwit Shinlapawittayatorn, Siriporn Chattipakorn

**Affiliations:** Cardiac Electrophysiology Unit, Faculty of Medicine, Chiang Mai University, Chiang Mai, Thailand

**Keywords:** ventricular fibrillation, induction, mechanism

## Abstract

Ventricular fibrillation (VF) is known as a main responsible cause of sudden cardiac death which claims thousands of lives each year.  Although the mechanism of VF induction has been investigated for over a century, its definite mechanism is still unclear.  In the past few decades, the development of new advance technologies has helped investigators to understand how the strong stimulus or the shock induces VF.  New hypotheses have been proposed to explain the mechanism of VF induction.  This article reviews most commonly proposed hypotheses that are believed to be the mechanism of VF induction.

## Introduction

Ventricular fibrillation (VF) was first described by Erichsen in 1842 [1].  It is known as a fatal cardiac arrhythmia that can cause sudden death.  This life-threatening VF has drawn the strong attention of a number of investigators for over a century.  The study of VF induction can be traced back to the original Ludwig and Hoffa study in 1850 in which they used a strong faradic current to induce VF. However, it was not until 1940 that Wigger and Wegria established the fundamental work which demonstrated that VF could be induced when a strong premature stimulus was applied during a certain period of the cardiac cycle [2].    This period is known as the “vulnerable period”, a period which corresponded to a portion of the T-wave of the surface electrocardiogram. The finding of VF induction by a strong stimulus delivered during the vulnerable period has allowed many investigators to advance the understanding of its mechanism.  Although many theories have been proposed as the fundamental mechanism of VF induction, none is universally accepted.  Current debates are discussed base on whether (1) reentrant  [3-5] or (2) focal pattern  [6-8]  that is responsible for VF induction after the shock.  In this review, four commonly proposed hypotheses are presented.

## The non-uniform dispersion of refractoriness hypothesis

It is known that membrane potential differences always exist in the intact heart during systole and diastole.  This is a result of the unequal levels of the resting membrane potentials as well as the depolarization potentials in myocardial cells and pacemaker cells   [9-11].  This heterogeneity is known as the “dispersion of refractoriness” of the tissues  [9,12] and already appears throughout the cardiac cycle of a normal heartbeat.  It is well accepted that to induce VF, the strength of a premature stimulus must be sufficiently strong (i.e. a threshold level) and be delivered during the vulnerable period  [2,13]. This shock strength is known as the VF threshold (VFT)  [14,16]. It is believed that VF is induced when the amount of heterogeneity or dispersion of refractoriness reaches a level that allows unidirectional block to occur, leading to reentry and fibrillation  [9,17].  This concept was supported by the discovery that VF is most likely to occur when the dispersion of refractoriness increases  [4,5]  [18,19].  By preconditioning the heart in a various setting to set up a non-uniform dispersion of refractoriness such as by slowing heart rate, stimulating cardiac sympathetic nerve, or by causing ischemic myocardium, many VF induction studies have demonstrated that the VFT was decreased when the degree of dispersion of refractoriness increased  [4,14]  [20].   Because of the heterogeneity of refractoriness of cardiac cells in various regions on the heart, activations are generated by the stimulus in excitable areas which are blocked unidirectionally when they encountered areas of greater refractoriness, leading to reentry and eventually VF.  Therefore, in this hypothesis, VF occurs by reentry caused by non-uniform dispersion of refractoriness.

It has been shown that responses of cardiac tissues to the shock can be in one of three categories, depending on the state of the myocardium at the time of the shock  [21-23].  First, the action potential duration will be extended if the shock is delivered to effective or relatively refractory tissue  [12,24,25].   This is commonly known as the “graded response”.  Second, the action potential will not be affected by the shock if it falls into the absolute refractory tissue.  And third, a new action potential will be created if the shock falls into the completely recovered tissue.  The degree of dispersion of refractoriness caused by a strong stimulus is mainly due to different responses of cardiac cells in different areas, resulting in the heterogeneity of refractory period extension in different cardiac cells throughout the heart  [18,26,27].

The vulnerable period is known to have a high degree of dispersion of excitability during the cardiac cycle  [9]. Hence, when a premature stimulus is applied to the heart during the vulnerable period, the response of the myocardium to the shock creates an even greater dispersion of refractoriness because the cardiac myocardium is irregularly excitable during that period, facilitating reentry and resulting in the initiation of VF  [4,19,28,29].  Since a certain amount of heterogeneity exists in the normally functioning heart, the differences between this amount of preexisting heterogeneity and the heterogeneity induced by the electrical stimulus required for VF induction (i.e. at VFT) is sometimes considered the margin of safety  [9].

Other groups of investigators, however, suggested that focal activation could initiate fibrillation due to the non-uniform dispersion of refractoriness hypothesis  [6-8].  In vitro studies have shown that abrupt differences in repolarization of adjacent cardiac cells were found at the site where the repetitive firing occurred (i.e. focal re-excitation),   [8] and these sudden repolarization differences have been considered to be a mechanism for VF induction  [8,30,31]. The role of premature stimulus delivered during the vulnerable period in the in vivo studies, however, is still controversial.  Both the increase in automaticity of the pacemaker fibers and reentry due to the unidirectional block have been demonstrated to be responsible for VF induction  [32-34].

##  The critical point hypothesis

It is known that the potential gradient created by the shock is very strong at sites close to the shocking electrode and is weaker at more distant sites  [35].  This gradient distribution, therefore, creates a non-uniform gradient field.  The critical point hypothesis is based on this fact and the fact that there are three possible responses of cardiac tissue when a premature stimulus is delivered to cells of different excitability: no response, graded response (i.e. refractoriness extension), and new activation.  This hypothesis states that the mechanism for VF induction is due to unidirectional block and unidirectional propagation of activation caused by those three different responses of cardiac tissues, leading to reentrant activation and, finally, VF  [21].  The difference between the critical point hypothesis and the non-uniform dispersion of refractoriness hypothesis is that the critical point hypothesis suggests the reentrant pattern as the sole mechanism for VF induction, whereas the latter could have either reentrant or focal excitation as the mechanism for VF induction  [21].

**Figure 1:**
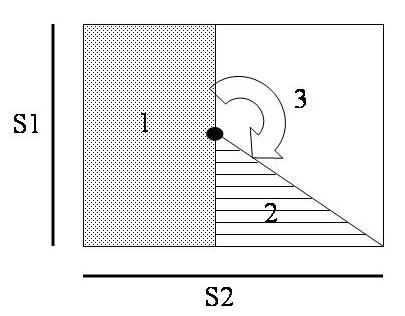
An illustrated diagram of the critical point formation.  S1 represents a pacing electrode.  S2 represents an electrode that delivers a strong stimulus.  When S2 is delivered at an appropriate time following an S1 stimulus, three areas of different cardiac responses are observed.  An Arrow represents the direction of reentry observed after the S2 shock is delivered.  A filled square represents the point where the critical point is formed.  See text for details.

The critical point hypothesis was first proposed mathematically by Winfree  [36] considering the heart as an excitable media, and was later demonstrated experimentally by Frazier et al  [21] in 1989.  In this experimental study, a strong premature stimulus (S2) was delivered to myocardium after a train of basic pacing stimuli (S1).  At an appropriate timing of the S2 delivery, they found the 3 types of myocardial responses to the S2 in 3 distinct regions (Figure 1).  First, the new activation created by the S2 shock arose at the recovered tissues, close to the S1 electrode, and was ready to propagate toward the excitable region (area 1, Figure 1).  (2) At the region far from the S1 electrode but close to the S2 electrode, the tissues were in their relative refractory period at the time the premature stimulus was delivered.  The S2 shock was strong enough to create a graded response, prolonging the refractoriness of the tissues in that area (area 2, Figure 1).  As a result, the new activation could not propagate through it.  (3) At the region farthest away from the S1 electrode (area 3, Figure 1), the S2 shock had no effect on the tissues in this whole area because the tissues close to the S2 electrode were still in their refractory period and not excitable and the S2 shock was too weak to create any response in the tissues far from it.  However, the myocardium in this region had sufficiently recovered in time to be excited by the activation front which propagates from the directly excited region.  This activation front could then reenter the area 2 and return to the area 1 again, since these cardiac tissues were already excitable.

The activation front would circle around the point where the three different cellular-response regions met (dark circle in  Figure 1).  This reentrant activation front could continue if the pattern of refractoriness of myocardium were maintained, or could be interrupted if the excitable pattern was changed.  It is important to note that in the critical point hypothesis, the angle between the S1 and S2 stimulus must be greater than zero to create the critical point for reentry  [21].  This reentrant activation front could continue if the pattern of refractoriness of myocardium were maintained, or could be interrupted if the excitable pattern was changed.  The formation of a critical point was thought to generate fibrillation in both VF induction by a premature stimulus and failed defibrillation  [37-40]. However, reentry is not always the pattern observed during VF induction or failed defibrillation.  Therefore, critical point formation may not be the sole mechanism of VF induction.

## The upper limit of vulnerability hypothesis

It is known that when a premature stimulus is given during the vulnerable period, there is a minimal strength needed to generate the inhomogeneity of excitability of cardiac tissues required to induce VF.  This strength is known as the VFT  [14].  When the strength of a premature stimulus is increased up to a level that VF is no longer induced at any time during the vulnerable period, this lowest strength that cannot induce VF is known as the upper limit of vulnerability (ULV)  [41]  [42]  The critical point hypothesis could be used to explain the existence of this ULV.  Since the formation of a critical point requires the cross point between the critical potential gradient and optimal excitable tissues, if this cross point is removed from the heart, the critical point will not be formed  [43].  It has been shown that as shock strength increases, distance of the critical potential away from the shocking electrode also increases  [35]. When the shock reaches the ULV strength, the critical value is off the heart.  Thus, no critical point is formed, and no VF is induced even when the shock strength is further increased  [43]  [44].

## The virtual electrode polarization hypothesis

This is the recent hypothesis proposed by Efimov to explain the induction of fibrillation  [45].  The concept of this hypothesis is similar to that of the critical point hypothesis, except that this hypothesis is not base on the potential gradient created by the shock delivered to the myocardium.  The findings that the shock can cause (1) depolarization or hyperpolarization of cardiac cells close to the shocking electrode, and (2) opposite polarization of cardiac cells in the region adjacent to (1) are the fundamental concept of this hypothesis.  When the optimal transmembrane potential gradient is generated in the region near the shocking electrode, reentry can be observed as activation propagates from depolarized tissues into hyperpolarized regions (*see figure 7 in reference 45*). This hypothesis is proposed to explain how fibrillating activation was observed after failed defibrillation (*see figure 11 in reference 45*).

Although reentry has been proposed in most hypotheses as the mechanism responsible for VF induction, recent VF induction studies in pigs have demonstrated different findings.  Chattipakorn et al have shown that following near ULV shocks, the first few post-shock activations arose on the epicardium in a focal manner before degenerating into VF  [46,47].  No reentry was observed in these studies. It has also been shown that ablation performed in the region where the early post-shock activation occurred could significantly decrease the ULV shocks  [48].  It is possible that focal activation observed in these studies is epicardial breakthrough resulting from transmural or endocardial reentry.  Further studies are under investigation to validate this hypothesis. Other mechanisms including the vortex theory and the mother rotor theory also have been proposed to be responsible for initiation and maintenance of VF  [49-51].  The definite mechanism, however, have yet to be revealed.

## Conclusion

Similar to defibrillation mechanism, the mechanism of VF induction is complicate.  Although its mechanism has been investigated for so many decades, how VF is induced is still debated.  Further studies of VF induction and defibrillation are essential since they will provide important information on the fundamental mechanism that can be used to improve the treatment and prevention of sudden cardiac death, which is mainly caused by VF in the future.
